# Development of a Mouse Model of Menopausal Ovarian Cancer

**DOI:** 10.3389/fonc.2014.00036

**Published:** 2014-02-26

**Authors:** Elizabeth R. Smith, Ying Wang, Xiang-Xi Xu

**Affiliations:** ^1^Department of Cell Biology, Sylvester Comprehensive Cancer Center, University of Miami School of Medicine, Miami, FL, USA; ^2^Department of Obstetrics and Gynecology, University of Miami School of Medicine, Miami, FL, USA

**Keywords:** ovarian cancer, epithelium, menopause, mouse models, ovarian follicles, pre-malignant lesions, Tp53

## Abstract

Despite significant understanding of the genetic mutations involved in ovarian epithelial cancer and advances in genomic approaches for expression and mutation profiling of tumor tissues, several key questions in ovarian cancer biology remain enigmatic: the mechanism for the well-established impact of reproductive factors on ovarian cancer risk remains obscure; cell of origin of ovarian cancer continue to be debated; and the precursor lesion, sequence, or events in progression remain to be defined. Suitable mouse models should complement the analysis of human tumor tissues and may provide clues to these questions currently perplexing ovarian cancer biology. A potentially useful model is the germ cell-deficient Wv (white spotting variant) mutant mouse line, which may be used to study the impact of menopausal physiology on the increased risk of ovarian cancer. The Wv mice harbor a point mutation in c-Kit that reduces the receptor tyrosine kinase activity to about 1–5% (it is not a null mutation). Homozygous Wv mutant females have a reduced ovarian germ cell reservoir at birth and the follicles are rapidly depleted upon reaching reproductive maturity, but other biological phenotypes are minimal and the mice have a normal life span. The loss of ovarian function precipitates changes in hormonal and metabolic activity that model features of menopause in humans. As a consequence of follicle depletion, the Wv ovaries develop ovarian tubular adenomas, a benign epithelial tumor corresponding to surface epithelial invaginations and papillomatosis that mark human ovarian aging. Ongoing work will test the possibility of converting the benign epithelial tubular adenomas into neoplastic tumors by addition of an oncogenic mutation, such as of Tp53, to model the genotype and biology of serous ovarian cancer. Model based on the Wv mice may have the potential to gain biological and etiological insights into ovarian cancer development and prevention.

## Introduction

Most ovarian cancers are epithelial-derived, and of the four major histological subtypes, serous ovarian cancer accounts for approximately 70% of the tumors (1–4). Serous ovarian carcinomas usually present as high-grade, with limited therapy options (5–7). Standard treatment regimens involve surgery to remove all visible disease, followed by a combination of taxane and platinum-based chemotherapy. Most patients who respond to first line chemotherapy will eventually relapse and die from drug-resistant disease. Despite intensive research and improvements in surgery and chemotherapy, the 5-year survival rate for ovarian cancer patients has languished around 30% for the past 30 years (5–7). This dismal survival rate attests to the urgency for a clear, more accurate understanding of basic ovarian cancer biology and etiology.

In the last several decades, great effort has been devoted to understanding ovarian cancer and the research has yielded significant knowledge and information about the biology and genetics of the disease (1–4). BRCA1 and BRCA2 mutations are associated with hereditary breast and ovarian cancers (1–4), which account for only a small fraction (estimated to be around 5–10%) of ovarian cancer cases. Recently, the Cancer Genome Atlas Project has provided a molecular profile of serous cancers (8): the tumor suppressor Tp53 is frequently mutated, but no other somatic mutation is consistently or frequently found. Nevertheless, Tp53 deletion alone is insufficient to induce epithelial tumors in mouse models (9–14). Thus, the molecular mechanism of ovarian serous cancer is not completely understood. In all the many types of ovarian tumor mouse models published so far, none reflects both the genetic (p53 mutation) and serous histology of human cancer.

Another key question in ovarian cancer biology related to reproductive etiology remains unanswered (1–4). Reproductive factors, such as increased parity and use of oral contraceptives, reduce the risk of ovarian cancers. Age and menopausal statues are even more important factors in ovarian cancer risk (1–4). Most ovarian cancers are diagnosed in menopausal women; fewer than 15% are diagnosed in women younger than 50 years of age, and the histological subtype of those cancers may not be epithelial but derived from germ cells or granulosa cells (15). The risk of ovarian cancer increases greater than fivefold during the peri-menopausal years (16–23).

In laboratory studies, few of the developed ovarian tumor models incorporate the epidemiological evidence that reproductive factors and age influence the risk of ovarian cancer. Consequently, the mechanism for the well-established impact of reproductive factors on ovarian cancer risk remains obscure and not well explored. Thus, a reasonably good model to understand the etiology of ovarian cancer should incorporate the genetics and the reproductive physiology of the disease, such as menopausal stage. Here, we discuss the development of a unique mouse model to study menopausal ovarian cancer.

## Ovarian Cancer Epidemiology and Etiology

Epidemiological evidence suggests that the risk of ovarian cancer associates with reproductive history and hormonal factors (16–23). Increased parity decreases the risk by 50% over nulliparity, as does oral contraceptive use for 5 years (17, 19, 20). The most significant risk factors for developing ovarian cancer are age and menopausal status (16–23). The majority of ovarian cancers are diagnosed in post-menopausal women in their late 50s and early 60s. The average age of diagnosis for sporadic ovarian cancer is about 63 years, although women with genetic or familial risk factors tend to be diagnosed at a younger age (average age of diagnosis is 54 years). Thus, it appears that age and menopausal status closely associate with ovarian cancer risk.

Several theories have been proposed to explain the epidemiological data associated with ovarian cancer risk. One idea holds that incessant ovulation, or the repeated wounding and subsequent proliferation that occur to repair the surface epithelium at the site of ovulation, results in mutations accumulating in the ovarian surface epithelial cells (24–26). Ultimately a tumor mass develops. This idea would explain the reduction of risk associated with pregnancy, extended breastfeeding, some oral contraceptive formulations, and early menopause, all of which reduce the number of ovulatory events.

Supported by the same epidemiological evidence, the gonadotropin stimulation hypothesis postulates that the surges of pituitary gonadotropins [including follicle stimulating hormone (FSH) and luteinizing hormone (LH)] that initiate each ovulation also stimulate the ovarian surface epithelium and induce cell transformation (20, 21). The speculated role of gonadotropins is also consistent with the fact that ovarian cancer occurs most frequently in post-menopausal women, when ovulation ceases yet plasma gonadotropins are elevated (21–23). However, since FSH and LH have unremarkable effects on growth of ovarian surface epithelial cells in culture (27–29), a direct effect of the hormones on ovarian epithelial transformation is unlikely to be sufficient. Thus, neither theory completely or satisfactorily explains the epidemiological observation of an association between ovarian cancer incidence and the menopausal transition.

A more recent idea posits that the depletion of ovarian follicles disrupts ovarian epithelial homeostasis and may be the true cause of an increased cancer risk in menopause (30). The idea that loss of ovarian function may underlie the link between reproductive factors and ovarian cancer was also proposed previously (31). The follicle depletion hypothesis explains the association between menopause and ovarian cancer risk, and can potentially unify “incessant ovulation” and “gonadotropin stimulation” as mechanisms. Specifically, incessant ovulation leads to the depletion of the ovarian reserve, which in turn leads to the increased level of gonadotropins that characterize menopause. Thus, the two theories explain the cause and consequence, respectively, of ovarian follicle depletion. The studies of a germ cell-deficient Wv mouse line provided basis for the follicle depletion theory (30, 31).

## Biology of Menopause

By the end of the reproductive age, germ cells and follicles are depleted from the ovaries and the ovulatory cycle ceases, resulting in menopause. Menopause is defined as the permanent cessation of menstruation resulting from depletion of germ cells and loss of ovarian follicular activity (32–34), and has become a woman’s health issue as a by-product of modern health advances and the extension of lifespan that occurred in the last century (32–34). The peri-menopausal period commences when the first features of menopause begin until at least 1 year after the final menstrual period, generally lasting an average of 5 years. In humans, the transition to menopause is a set of gradual changes, in which ovarian function, reproductive capacity, and hormonal status are altered long before menses stops completely. Menopause generally occurs between 45 and 55 years of age, and the symptoms vary among women.

Hormonal changes characterize the menopausal transition. In the normal reproductive ovary, following ovulation and release of the ovum, the follicle converts into a corpus luteum, where sex steroids, predominately estrogen and progesterone, are produced and released. The steroid hormones act to inhibit the release of FSH and LH. With the depletion of follicles and cessation of ovulation, estrogen and progesterone levels fall and normal feedback inhibition of FSH and LH release stops. As a result, FSH and LH reach highest serum levels in peri- and post-menopausal periods and remain elevated (32–34). These changes precipitate a number of menopausal-associated symptoms and disorders.

## Mechanisms for Menopause as a Risk Factor

Among the physiological changes associated with menopause, the ovarian tissues undergo morphological transformation, known as “ovarian aging” (25), and this is implicated in the high incidence of ovarian cancer that occurs during the peri-menopausal and immediate post-menopausal periods (30, 31). One feature associated with ovarian aging is the accumulation of ovarian morphological changes such as deep invaginations, surface papillomatosis, and inclusion cysts (35–37), which are thought by some to be the histological precursors of ovarian cancer (38–43). Presumably, acquisition of an oncogenic mutation (such as Tp53 mutation) in these proliferative ovarian epithelial cells would promote the development of ovarian cancer.

From the analysis of pre-cancerous ovarian tissues obtained from prophylactic oophorectomies, pre-neoplastic lesions and microscopic carcinomas were identified in the ovaries or fimbria of fallopian tubes from women with a family history of ovarian cancer or identified BRCA mutations (38, 44, 45). Several studies reported the increased ovarian morphological changes in high-risk ovaries (35, 37, 38, 46, 47), though some found negative results (48–50). In one analysis, we found that no significant increase in the presence of non-neoplastic ovarian morphological changes is associated with BRCA1/BRCA2 mutations (35). Rather, the frequency of these histological features, especially inclusion cysts, associates with age or menopausal status. We propose that ovarian morphological changes increase in the peri-menopausal period, and these histological features may promote the transformation of genetically compromised epithelial cells in the development of ovarian cancer. The results suggest age-dependent pre-neoplastic morphological changes may be a risk factor, and support the idea that ovarian aging-related epithelial morphological changes provide precursor cells that may transform upon acquisition of oncogenic mutation(s) (42).

The fallopian tube origin of ovarian cancer suggests that tubal epithelial cells from the normal fimbria, which envelops the ovary and contacts the ovarian surface, dislodge and seed, or implant on, the surface of the ovary (51–58). Inclusion cysts form by membrane engulfment. Likewise, transformed cells of the fimbria may shed and implant on the ovarian surface. The tumor that establishes appears to arise from the ovary but originates, in fact, from the fallopian tube. It may be that age and follicle depletion alter the receptivity of the ovarian surface to seeding by the fallopian fimbria epithelial cells, i.e., its ability to accept the fimbria cells, and also make it a more permissive substratum for engulfment or proliferation of the seeded cells. Thus, the idea of follicle depletion as a risk factor for ovarian cancer may also be adapted to the fallopian tube cell of origin model, in addition to that originally proposed considering only cancer derived from the ovarian surface and/or surface-derived inclusion cysts (30, 31). Additionally, follicle depletion may also encourage the proliferation of stromal epithelial cells of Müllerian origin, which have also been considered to be possible cells of origin of ovarian serous carcinomas (59, 60). The epithelial cells of both fallopian tube fimbria and extra-ovarian Müllerian glands may be responsive to the menopausal increase of gonadotropins.

## Mouse Models in Ovarian Cancer Research

In the past decade, a number of technical breakthroughs have led to the establishment of several mouse models as described briefly here. First, a genetically defined model of ovarian cancer was established by Orsulic and colleagues (13), in which mouse ovarian surface epithelial cells were isolated and transfected with defined genetic changes such as k-Ras, v-Akt, v-myc, etc. The cells were then re-implanted into the ovarian bursa of mice and malignant ovarian tumors developed. Using the MIS II R promoter, a mainly ovarian-restricted transcript, Connolly, Hamilton and colleagues developed the T-antigen transgenic line that develops malignant bilateral ovarian tumors (61). Presumably, T-antigen expression results in the inactivation of both p53 and Rb. Indeed, using adenoviral delivery of cre to ovaries of mice with floxed p53 and Rb, Flesken-Nikitin et al. demonstrated the development of malignant ovarian tumors when both p53 and Rb are deleted (11). Mice with conditional expression of K-ras and deletion of pten in ovarian surface epithelial cells were made and found to develop endometriosis and endometrioid carcinomas (62). Since both mutations are associated with endometriosis and endometrioid ovarian cancer in humans, this model appears to recapitulate the genotype and histomorphology of the human disease. Another mouse model of endometrioid carcinomas was established by combining beta-catenin activation and pten loss (63). Based on the understanding that the majority of serous ovarian cancer may be derived from fallopian tube fimbria, the reproductive tract tumor models were produced by targeting SV40T using the promoter of the mouse oviduct-specific glycoprotein (OGP) (64). In another study, fallopian tube-derived tumors were produced by Amhr2-Cre mediated deletion of pten and Dicer (65). Likely there are additional ovarian cancer animal models that are not mentioned here (66–69).

However, the modeling of genotype and phenotype of human serous cancer has not been successful. Although p53 mutation is the only common genetic mutation in ovarian cancer (8), p53 null mice do not develop ovarian cancer. When p53 null ovaries were transplanted into wild type mice to allow prolonged aging, the tumors that developed were of granulosa rather than epithelial origin (9). In several recent studies, concomitant inactivation of Tp53 and BRCA1 produced leiomyosarcomas, which likely originated from the ovarian bursa (10, 12–14). Further investigation of these animal models should lead to a better, more thorough understanding of ovarian cancer development. Nevertheless, none of these models has components related to the etiology of ovarian cancer. Also, few investigations on early lesions or cells of origins were reported in these ovarian tumor models.

To investigate reproductive factors, mouse models that mimic or incorporate menopausal biology may be useful. Most female mammals, except for humans, live only a relatively short time after ceasing reproduction, and normal rodents or other animals do not adequately model the menopausal state (32). In the laboratory setting, surgical removal of ovaries is used to mimic menopause on the physiology. Another method is to kill germ cells and ovarian follicles using toxins such as such as busulfan and 4-vinylcyclohexene diepoxide (70–72). These “menopausal mouse models” may be useful for some purposes, for example, to investigate breast tumor xenografts under menopausal conditions and to study chemical-induced breast carcinogenesis (73, 74). Mutant mice that contain gene mutation affecting ovarian function were also suitable to investigate ovarian cancer. A notable mouse model of restricted BRCA1 deletion in granulose cells was produced to investigate the association between menstrual cycle and ovarian cancer risk (75, 76). Mice with FSH receptor knockout were reported to exhibit some phenotype of ovarian failure and have been proposed as a potential model of menopause (77). In this article, we highlight the use of a natural mutant mouse line, the white spotting variant (Wv) mouse, to model menopause and associated ovarian cancer risk. In the Wv females, the ovarian follicles are gradually depleted early in life because of a reduced c-kit activity and resulted oocyte reserve, and the mice mimic the phenotypes in both the cause (ovarian follicle depletion) and many consequences (such as changes in heart, bone, lipids, ovarian epithelia) of menopause (78, 79).

## The Wv Germ Cell-Deficient Mouse Models

**Figure 1:**
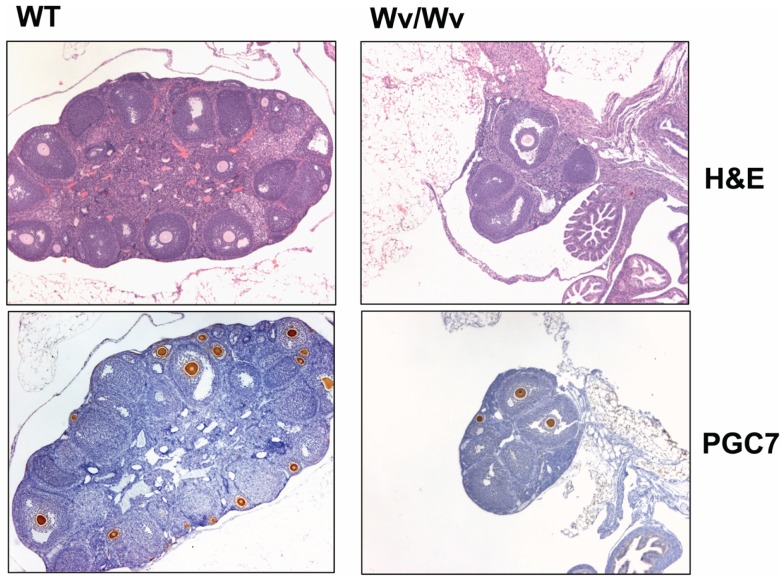
**Germ cell deficiency in Wv ovaries**. Ovaries from 6-week-old wild type and Wv/Wv littermates were harvested and subjected to histological analysis. PGC7 staining was used as a marker for germ cells and follicles (86, 87). Wild type ovaries contain abundant PGC7-positive germ cells and follicles of various developmental phases, and in particular, germ cells and primary follicles are found immediately beneath the surface. Wv/Wv mutant ovaries are smaller and contain a greatly reduced number of germ cells.

The Wv mice harbor a point mutation in the kinase domain of the c-kit gene, resulting in developmental defects in germ cells, pigment-forming cells, red blood cells, and mast cells in homozygous mutant mice (68–83). The Wv/Wv mice have a similar lifespan as wild type, are sterile, white-coated with black eyes, and predisposed to ovarian neoplasms (84). The Wv/Wv mice contain less than 5% of the normal number of oocytes at birth and the remaining germ cells are depleted by about 8 weeks of age (Figure 1). Consequently, ovulation ceases to occur and an increase in pituitary gonadotropins follows (85). Compared to wild type littermates, in which ovaries contain a large number of follicles at various developmental stages, Wv ovaries are depleted of follicles by 2–3 months of age (Figure 1). Ovarian surface epithelial dysplasia and tubular adenomas develop in Wv/Wv mice (79, 85). The Wv mice appear to model several aspects of post-menopausal biology, including a long post-reproductive lifespan, increased serum gonadotropins, decreased sex steroids, and physiological changes, such as decreased bone density, elevated serum cholesterol, and altered cardiac function (78).

**Figure 2:**
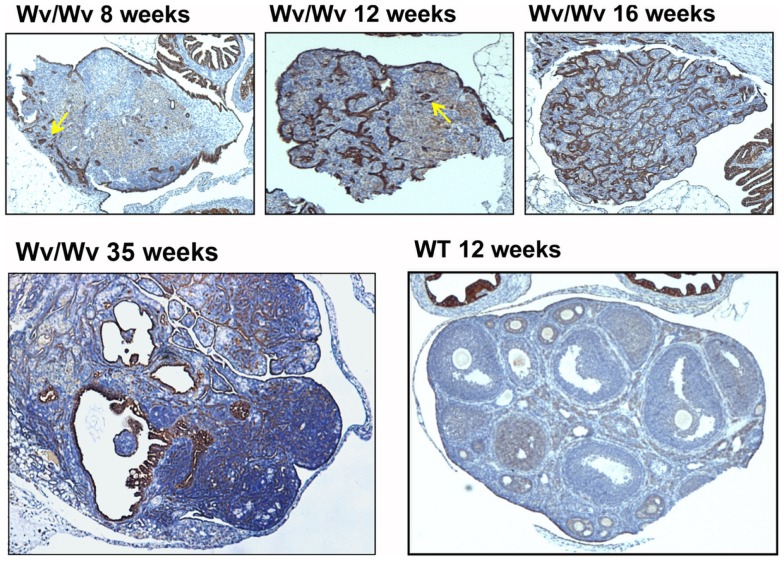
**Epithelial ovarian tumors in Wv/Wv mice**. Ovarian tissues were harvested and subjected to histological analysis. Cytokeratin-8 staining was used as a marker for epithelial cells. Representative ovaries are shown for 8-, 12-, 16-, and 35-week-old Wv/Wv mice, indicating the progressive increase in epithelial lesions. In comparison, a wild type ovary from a 16-week-old littermate has a single layer of cytokeratin-8-positive ovarian surface epithelium. The arrows in the two left panels indicate the putative lesions that developed from rete ovarii.

The ovarian lesions in the Wv mice distribute throughout the ovarian stroma, and are known as stromal tubular adenomas (85). The contiguous connection to ovarian surface epithelium is evident (Figure 2), and is especially pronounced in early ovarian lesions from younger (7–10 weeks) mice when lesions begin to develop (79). The majority if not all the tubular adenomas in Wv/Wv ovaries appear to be derived from ovarian surface epithelial cells. However, rete ovarii structure is also very prominent in Wv ovaries. At 4 months, epithelial lesions permeate the entire ovary, and rete ovarii appear to form distinct lesions (Figure 2, arrow). At 8 months of age, the Wv ovarian tumor is extensive, and surface versus rete ovarii epithelia are no longer distinguishable. The majorities of the lesions either exhibit inclusion cyst-like structures or resemble surface deep invaginations/papillomatosis (Figure 2) (79).

## Potential Development of the Wv Mice to Model Menopausal Ovarian Cancer

The germ cell-deficient Wv mutant mouse line mice may be explored to gain additional understanding and verification of the impact of menopausal physiology on the increased risk of ovarian cancer.

**Figure 3:**
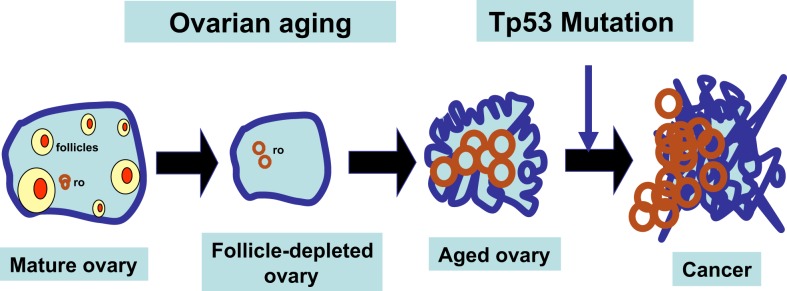
**Working model for follicle depletion and ovarian aging in ovarian tumorigenesis**. A schematic illustration of the consequences of follicle depletion in the development of ovarian tumors is presented. Upon depletion of ovarian follicles, ovarian surface and rete ovarii (ro) epithelia undergo remodeling and morphological changes. Wv ovarian epithelial tumors may be derived from both surface and rete ovarii (ro). Genetic mutations, such as Tp53 mutation, will promote the benign epithelial lesions to develop into malignant tumors.

Tp53 deletion, alone or in combination with other genetic changes, does not seem to produce ovarian epithelial tumors in mouse models. Since mutations in Tp53 that result in accumulation of mutated Tp53 protein occur frequently in ovarian cancer and are more relevant than deletion (8, 88), it may be possible to add the Tp53 mutation in the Wv ovarian tubular adenomas to test if Tp53 mutation can convert the benign epithelial tumors to malignant adenocarcinomas (Figure 3). If successful, such a model may mimic the development epithelial ovarian cancer in both genetic and reproductive aspects. Using the Wv mice, we are currently performing experiments to determine if adding a Tp53 point mutation in the epithelial cells of the Wv ovarian tumor generates a malignant tumor that resembles ovarian cancer.

By deleting a transcription stop signal in the floxed Tp53 mutant (88) in ovarian surface epithelial cells through injection of adenovirus expression Cre, we predict that the model mimics both reproductive factors (postmenopause) and genetic mutation (Tp53). Preliminary studies indicate that these Wv/Wv:p53(R172H) (fl/fl):Adv-Cre ovarian epithelial tumors appear malignant. We are currently characterizing in more detail these mouse ovarian epithelial models and expect to report these findings in the near future.

## Possible Strategies for Delaying Menopause and Ovarian Cancer Risk Reduction

The neoplastic ovarian tumor models following addition of Tp53 mutation in the benign Wv tumors may be used to explore several questions regarding the etiology and possible preventive strategies for ovarian cancer. Several potential preventive approaches, such as inhibition of cyclooxygenases and use of progestin to mimic oral contraceptive usage, have been proposed and can be tested in Wv mouse models.

Genetic suppression of cyclooxygenase 2 produced a significant alleviation of ovarian lesions in the Wv/Wv:Cox-2 (±) ovaries analyzed, although the degree to which the tumor phenotype was suppressed varied greatly (79). Hemizygous reduction of the Cox-2 gene resulted in a complete or partial rescue from the epithelial adenoma phenotype. Thus, a reduction in Cox-2 gene dosage rescued the ovarian epithelial morphological alteration, but deletion of both copies was less sufficient in reversing the adenoma phenotype. Reducing the Cox-2 gene dosage on ovarian tumor phenotype can be achieved by using pharmacological agents. Thus, cyclooxygenase inhibitors are able to prevent ovarian epithelial morphological transformation and tumor phenotypes. Inhibition of both Cox-1 and Cox-2 with indomethacin is more effective than inhibition of Cox-2 alone with celebrex. When indomethacin was given for a period of 1 month to Wv/Wv mice at 3 months of age when ovarian tumors were already established, the tumors were not reduced compared to controls, suggesting inhibition of cyclooxygenases prevents the development of ovarian tumors but has no suppressive effect on established tumors (79). Furthermore, inhibition of Cox-1 was superior to inhibition of Cox-2, and inhibition of Cox-1 reduced the development of ovarian adenomas in Wv mice by delaying ovarian follicle maturation and thus depletion (89) occurs. The conclusions of these studies are consistent with the notion that the ovarian follicle depletion, rather than ovulation and gonadotropin stimulation, is a major determinant of an increased ovarian cancer risk in menopause. The experimental results provide explanation for the epidemiological observations that use of non-steroidal anti-inflammatory drugs (NSAIDs) reduce ovarian cancer risk (90–96). Inhibition of Cox-1 and Cox-2 may have different mechanisms. Cox-1 inhibition may delay follicle depletion and ovarian cancer risk. Cox-2 inhibitors may reduce the cancer promoting activity of the inflammation-like ovulatory processes that are stimulated by gonadotropins (95, 96). The mechanism predicts that use of NSAIDs may be more effective in reducing the risk of ovarian cancer in pre-menopausal compared to post-menopausal women, since Cox-1 inhibition can delay ovarian follicle depletion (89). In post-menopausal women, inhibition of Cox-2 may slow epithelial remodeling and thus still reduce ovarian cancer risk.

Because endogenous hormones play a major role in the risk of breast, endometrial, and ovarian cancer, the impact on risk for oral contraceptives and hormonal therapy given at about the time of menopause has been a major concern (97, 98). Numerous studies provide insights into cancer risk associated with use of these preparations. Generally, use of oral contraceptives reduces ovarian cancer risk (19, 99). Many studies attribute the preventive effect on its suppression of gonadotropin level and ovulation. Also, this risk reduction may differ between pre- and post-menopausal women. Recent studies suggest that prolonged oral contraceptive pill use provided a greater protective effect against pre-menopausal ovarian cancer than against post-menopausal cancer (100). Furthermore, suppression of pituitary gonadotropin release with hormone replacement therapy may not reduce ovarian cancer risk in post-menopausal women (97, 100). These findings substantiate that intact ovarian function may be an important determinant of ovarian cancer risk, and the timing of progesterone administration may differentially alter its preventive capacity depending upon follicle reserve and menopausal status. The Wv mouse model will be useful in experiments to test the suppressive activity of progesterone/progestin on gonadotropin levels and the role of increased gonadotropins on ovarian tumorigenesis.

## Conflict of Interest Statement

The authors declare that the research was conducted in the absence of any commercial or financial relationships that could be construed as a potential conflict of interest.
